# Navigating the Complexities of HIV Vaccine Development: Lessons from the Mosaico Trial and Next-Generation Development Strategies

**DOI:** 10.3390/vaccines13030274

**Published:** 2025-03-05

**Authors:** Victor Abiola Adepoju, Donald C. Udah, Okechukwu Innocent Onyezue, Qorinah Estiningtyas Sakilah Adnani, Safayet Jamil, Mohammed Nadir Bin Ali

**Affiliations:** 1 Department of HIV and Infectious Diseases, Jhpiego (An Affiliate of Johns Hopkins University), Abuja 900911, Nigeria; 2 JSI Research & Training Institute Inc. (JSI), Abuja 900911, Nigeria; donald.udah@gmail.com; 3 Department of HIV Prevention and Community Directorate, APIN Public Health Initiatives, Abuja 900911, Nigeria; ozueinnoson@gmail.com; 4 Department of Public Health, Faculty of Medicine, Universitas Padjadjaran, Bandung 40161, West Java, Indonesia; qorinah.adnani@unpad.ac.id; 5 Department of Public Health, Daffodil International University, Dhaka 1216, Bangladesh; safayet241-41-019@diu.edu.bd; 6 Department of Public and Community Health, Faculty of Medicine and Health Sciences, Frontier University Garowe, Puntland 00252, Somalia; 7 Department of Computing and Information System, Daffodil International University, Dhaka 1216, Bangladesh; drnadir@diu.edu.bd

**Keywords:** HIV, Mosaico, broadly neutralizing antibodies (bNAbs), vaccine development, clinical trials, virus-like particles, SOSIP trimers, mRNA technology, AI-driven vaccine design, T cells, adjuvants

## Abstract

**Background/Objectives:** The development of an effective HIV vaccine has faced persistent challenges, as evidenced by the recent discontinuation of the Mosaico phase 3 trial. This study aims to critically examine the obstacles encountered in HIV vaccine development, with a focus on the Mosaico trial, which tested the Ad26.Mos4.HIV vaccine among 3,900 participants across multiple countries. We also explore emerging vaccine technologies and their potential in overcoming these challenges, while reflecting on lessons from previous trials to inform future strategies. **Methods:** We reviewed the Mosaico trial’s approach, which involved testing the efficacy of the Ad26.Mos4.HIV vaccine. We compared the outcomes of the Mosaico trial with other major HIV vaccine trials, including HVTN 702, Imbokodo, and RV144. We explored the limitations of the immune responses elicited by the Mosaico vaccine. The review focused on the generation of broadly neutralizing antibodies (bNAbs) and the challenges related to antigenic diversity and B-cell engagement. Emerging vaccine technologies, such as virus-like particles (VLPs), nanoparticles, SOSIP trimers, and mRNA platforms, were also analysed for their scalability, immune durability, and potential to advance HIV vaccine development. **Results:** The Mosaico trial was discontinued due to insufficient efficacy in reducing HIV acquisition, primarily due to the inability of the vaccine to induce bNAbs, which are crucial for targeting the diverse HIV-1 strains. A major challenge was the inadequate engagement of germline B-cell precursors, compounded by the antigenic diversity of the virus. The analysis showed that emerging vaccine platforms, such as VLPs, nanoparticles, SOSIP trimers, and mRNA-based approaches, hold promise but present challenges related to scalability and the durability of immune responses. The role of T cells and adjuvants in enhancing vaccine efficacy was also highlighted as critical for integrating both humoral and cellular immunity. **Conclusions:** The Mosaico trial, as well as other major HIV vaccine trials, underscores the need for a multi-pronged approach that incorporates both antibody and T-cell responses to tackle the complexities of HIV-1. Future efforts in HIV vaccine development must focus on inducing bNAbs, generating robust T-cell responses, and utilizing scalable vaccine platforms. The integration of artificial intelligence (AI) into vaccine design offers new opportunities to optimize immunogenic targets, which could significantly improve the potential for durable and broad immune protection. The development of a successful HIV vaccine by 2030 is achievable but relies on leverage on advanced technologies including artificial intelligence, innovation and insights from past trial data.

## 1. Introduction

The recent failure of the Mosaico phase 3 trial marks a significant setback in the ongoing quest for a safe and effective HIV vaccine [1]. The Mosaico trial enrolled 3900 cisgender men and transgender individuals aged 18–60 years across eight countries, including the U.S., Argentina, and Spain. Participants were randomized 1:1 to receive either a series of four doses of the investigational vaccine (Ad26.Mos4.HIV with Clade C and Mosaic gp140 adjuvanted by aluminum phosphate) or a placebo over 12 months. Ad26.Mos4. HIV is a viral vector-based vaccine that expresses two mosaic Env antigens and two mosaic Gag-Pol antigens, which differ from earlier versions that included only one Env antigen [2]. Despite being well-tolerated, the interim analysis of the trial results by the Data and Safety Monitoring Board revealed no significant difference in HIV acquisition rates between the vaccine and placebo groups, leading to the discontinuation of the trial [2]. The failure of the Mosaico trial underscores the inherent bottlenecks and complexities in developing an HIV-1 vaccine, especially the challenge of inducing a protective immune response capable of preventing HIV infection. To a large extent, this failure is due to a lack of induction of bNAbs; notwithstanding, it is important to further explore the reasons why bNAbs were not induced and identify strategies that could be successful in future trials [3]. The Mosaico vaccine, based on adenovirus 26 vectors with a gp140 protein boost, failed to induce bNAbs largely due to the inability of the immune system to overcome the virus’s envelope diversity and immune evasion tactics, such as glycan shielding [3]. These design limitations hindered the vaccine’s ability to target conserved epitopes necessary for bNAb production [4]. Inducing broadly neutralizing antibodies (bNAbs) is crucial in HIV vaccine development due to the high genetic variability of the virus, particularly in its envelope glycoproteins (gp120 and gp41) [4]. These bNAbs can target conserved regions of the virus and effectively neutralize multiple strains. Given the rapid mutation rates of HIV, bNAbs offer the possibility of lasting protection by binding to stable viral epitopes and preventing viral entry into cells. Recent advancements in artificial intelligence (AI) and machine learning are revolutionizing vaccine design, powering the prediction of immunogenic epitopes, and the optimization of vaccine constructs [5] that offer new hope in the face of persistent challenges. For instance, AI has been used to optimize immunogen design by predicting epitope structures, resulting in enhanced vaccine target selection through neural networks and predictive analytics [6]. Machine learning accelerates vaccine development by identifying B and T cell epitopes, predicting antigen interactions, and refining immunogen constructs, which facilitates faster, more accurate vaccine target selection and reduction in development timelines [7]. This article aims to critically analyze the Mosaico trial, provide insights into the challenges faced, and debate potential strategies and advancements to address these challenges in HIV vaccine development.

## 2. Lack of bNAbs

The primary shortcoming of the Mosaico trial was its failure to induce bNAbs, which are paramount in neutralizing diverse HIV-1 strains [3]. One explanation for this outcome could be the failure of the vaccine to effectively target germline precursors of bNAbs [5]. The Mosaico vaccine, although designed to elicit bNAbs, primarily induced non-neutralizing antibodies and weak T-cell responses. This highlights the difficulty in precisely guiding the immune system toward the desired broadly neutralizing response. The primary reason is that nNAbs bind to epitopes that do not prevent viral entry but may facilitate antibody-dependent cellular cytotoxicity (ADCC) [8]. Conversely, bNAb production requires engaging specific germline B-cell precursors, a mechanism not effectively activated by the Mosaico vaccine design [8]. Emerging studies have highlighted the critical role of germline-targeting immunogens in driving the maturation of bNAbs, suggesting that precision in engaging specific B-cell lineages could significantly enhance vaccine efficacy [9,10]. Moreover, the process of germline targeting is complex, and it has been challenging for scientists to fully understand the specificities of germline precursors of bNAbs. The antigenic diversity of HIV-1 strains is another potential contributory factor for the lack of bNAb induction in the Mosaico trial since HIV-1 is characterized by a high mutation rate, leading to the emergence of diverse viral strains that can escape immune surveillance [8]. One of the most problematic tasks in HIV vaccine research is the development of a vaccine that can elicit bNAbs capable of neutralizing a wide range of HIV-1 strains. A deeper understanding of the intricacies of B cell ontogeny would undoubtedly facilitate strategies to precisely guide the immune response towards broadly neutralizing specificities. The inability to effectively target the rare germline precursors of bNAbs results from the challenge of designing immunogens that specifically engage and drive their maturation toward bNAb production [9]. A deeper understanding of the association between the induction of bNAbs and viral diversity is crucial for designing more effective HIV vaccines.

## 3. Emerging Approaches in HIV Vaccine Development

Cutting-edge technologies that utilize artificial intelligence and machine learning in vaccine design, optimization, and prediction of immunologic epitopes, coupled with the emerging approaches discussed below, offer renewed hope in the pursuit of an effective HIV vaccine [5]. One of these approaches is the virus-like particles (VLPs) and nanoparticles. VLPs have been studied extensively as a promising alternative for inducing bNAbs in HIV vaccine development [11,12]. Ferritin-based nanoparticles have shown considerable promise in vaccine development, especially in inducing bNAbs against diverse viral strains [13]. Ferritin-based nanoparticles present multiple copies of antigens in a symmetrical structure, which enhances B-cell receptor engagement, leading to stronger immune activation. By presenting diverse viral epitopes on their surface, these nanoparticles mimic viral particles, which improves their ability to stimulate bNAbs [13]. Preclinical studies using ferritin nanocages as platforms for HIV vaccines have elicited broad immune responses, with some achieving neutralization against up to 60% of circulating HIV strains due to efficient epitope display [13]. Their stability and multivalent antigen presentation make them a promising option for HIV vaccine development, as demonstrated in studies using ferritin-bound mRNA vaccines encoding HIV-1 Env trimers [14]. For example, ferritin nanoparticles have been used in preclinical studies to present HIV-1 Env trimers, influenza antigens, and Epstein-Barr virus antigens, all demonstrating robust neutralizing antibody responses [13]. Two-component nanoparticles are some of the recent advances in VLPs that have demonstrated potential in eliciting bNAbs against diverse HIV-1 strains [15,16,17]. These innovative approaches warrant further investigation in future vaccine trials. That notwithstanding, the development of nanoparticles and VLPs as vaccine platforms is not without their challenges, such as the optimization of their size, shape, and immunogenicity to improve bNAb induction. However, there is a need to further explore the scalability and manufacturing of VLPs and nanoparticles for large-scale vaccine production.

## 4. SOSIP Trimers and mRNA Technologies

SOSIP trimers stabilize HIV-1 Env into its native trimeric form by introducing a disulfide bond between gp120 and gp41 and modifying the gp41 structure to enhance its stability. These modifications have been shown to induce bNAbs in animal models [18]. Some of the challenges faced by the Mosaico trial can potentially be addressed through the use of SOSIP trimers as immunogens in HIV vaccine development. SOSIP trimers have been stabilized to mimic the native conformation of the HIV-1 Env trimer, inducing bNAbs in preclinical trials. One study involving infant macaques demonstrated the elicitation of bNAbs through SOSIP vaccination [18]. These results highlight the potential of SOSIP trimers in advancing HIV vaccine strategies. Moreover, the potential instability of SOSIP trimers and the need to identify the most immunogenic SOSIP trimer variants could still limit this approach. In addition, COVID-19 vaccine development has shown the success of mRNA technology, thereby ushering new avenues and opportunities for HIV vaccine development [14]. These technologies offer potential platforms for creating effective HIV vaccines that induce bNAbs and necessitate further investigation. However, the applicability of mRNA technology to HIV vaccine development also presents challenges, such as the need to overcome potential mRNA instability and optimization of delivery methods [14]. Other challenges include the high mutation rate of the virus, which complicates targeting conserved epitopes, and the instability of SOSIP trimers during storage. The high mutation rate of HIV poses a challenge for mRNA vaccines because the rapidly changing sequence of the virus complicates immunogen design. Furthermore, while mRNA vaccines have shown great promise in the context of COVID-19, their application to HIV vaccine development faces unique hurdles, such as the need to encode complex envelope glycoproteins and ensure their proper conformation and glycosylation for optimal immunogenicity.

## 5. Role of T Cells and Adjuvants

In addition to bNAbs, T cells play a critical role in the immune response against HIV [19]. A recent study showed that vaccines inducing both antibody and T-cell responses provided greater protection against HIV infection [19]. This evidence indicates that a vaccine that focuses solely on bNAbs, as in the Mosaico trial, may be insufficient to achieve breakthrough efficacy. Future strategies must integrate robust T-cell responses to complement bNAb induction. Adjuvants have been shown to enhance T-cell responses by improving antigen presentation and modulating T-B interactions, which are crucial for affinity maturation of antibodies and memory cell formation [20,21]. Future HIV vaccine research should, therefore, not only incorporate T cell responses but also optimize adjuvants in order to improve vaccine efficacy. Adjuvants can enhance the immune response by modulating the physical and functional properties of induced antibodies [20,21], but selecting the most appropriate adjuvants for HIV vaccines requires a deeper understanding of the immunological mechanisms involved in protection against HIV-1 infection. Examples of adjuvants used in HIV vaccine development include MF59 (an oil-in-water emulsion), AS01B (containing saponins), and Alhydrogel, all of which enhance immune activation by increasing antigen uptake and presentation [22].

## 6. Comparative Analysis of Key HIV Vaccine Trials

To better understand the complexities of HIV vaccine development and the lessons learned from past failures, we compare the key features and outcomes of the Mosaico trial with other notable HIV vaccine trials, including HVTN 702, Imbokodo, and the partially successful RV144 trial (Table 1).

The failure of the Mosaico trial [2] is part of a broader pattern in HIV vaccine research, as previous trials like HVTN 702 [23] and Imbokodo [24] also struggled to generate sufficient protective immunity. Differences in target populations and immunization regimens across the trials played a significant role in the outcomes. Mosaico, targeting MSM and transgender individuals, and Imbokodo, targeting women in Sub-Saharan Africa, faced the challenge of high exposure risk and viral diversity. These trials were unable to induce the necessary breadth of immune response, particularly in generating durable neutralizing antibodies. In contrast, RV144 [25], although modestly successful, involved a more general population and induced both humoral and cellular immune responses, with partial protection attributed to non-neutralizing antibodies and CD4+ T cell responses. This suggests that the combination of humoral and cellular immunity, as seen in RV144, may be crucial in future HIV vaccine strategies.

These insights emphasize the importance of tailoring immunization strategies and vaccine platforms to specific populations while ensuring robust, broad, and durable immune responses. Lessons from the trials indicate that future vaccines may need to optimize both antibody and T-cell responses, as well as adapt to the unique demographic and geographic characteristics of target populations.

## 7. Conclusions

The failure of the Mosaico trial underscores the urgent need for continued innovation, investment, and collaboration in HIV vaccine research. Key priorities for future research include targeting germline precursors of bNAbs, enhancing T-cell responses, and exploring combination vaccine strategies that leverage new technologies like mRNA and nanoparticle platforms. Challenges faced by the trial, such as inducing bNAbs and the complexity of HIV-1 antigenic diversity, emphasize the need for deeper understanding and innovative approaches to overcome these obstacles. Emerging technologies like VLPs, nanoparticles, SOSIP trimers, and mRNA vaccines present potential platforms for future HIV vaccine development, but they also come with their own challenges that must be addressed. The failure of the Mosaico trial underscores the need for multi-pronged vaccine approaches that engage both humoral and cellular responses. Future HIV vaccines must focus on bNAbs, T-cell activation, and scalable platforms like mRNA technologies to overcome the challenge of viral diversity. Furthermore, it is important to incorporate both antibody and T cell responses in HIV vaccine design. Based on the cumulative evidence from these trials, it appears that a successful HIV vaccine will likely require a multi-pronged approach, combining the induction of bNAbs, T cell responses, and potentially innate immune activation. This integrated strategy, informed by a deeper understanding of HIV immunology and cutting-edge technological advancements, offers the best hope for overcoming the persistent challenges in HIV vaccine development. The Mosaico, Imbokodo, HVTN 702, and RV144 trials offer invaluable insights into the challenges and opportunities in the pursuit of an effective HIV vaccine. By critically analyzing the findings from these trials and leveraging advancements in immunology and vaccine technology, the scientific community could finally develop a successful HIV vaccine, bringing us closer to ending HIV by 2030.

## Figures and Tables

**Table 1:** Comparative overview of key HIV vaccine efficacy trials: Mosaico, HVTN 702, Imbokodo, and RV144.

Trial Name	Mosaico (HVTN 706) [2]	HVTN 702 (Uhambo) [23]	Imbokodo (HVTN 705) [24]	RV144 [25]
Start Year	2019	2016	2017	2003
Target Population	3900 cisgender men and transgender people who have sex with cisgender men and/or transgender people, who are at increased vulnerability to HIV acquisition in the Americas, Europe (Argentina, Brazil, Italy, Mexico, Peru, Poland, Spain, and the United States)	5407 men and women in South Africa	2636 women aged 18–35 in Sub-Saharan Africa (Malawi, Mozambique, South Africa, Zambia, and Zimbabwe.)	16,402 heterosexual men and women in Thailand
Vaccine Platform	Ad26.Mos4.HIV vector with gp140 protein boost	ALVAC-HIV (recombinant canarypox) vector + MF59-adjuvanted gp120 boost	Ad26.Mos4.HIV vector with clade C gp140 protein boost	ALVAC-HIV (canarypox vector) + AIDSVAX B/E (gp120 boost)
Primary Endpoints	Prevention of HIV infection	Prevention of HIV infection	Prevention of HIV infection	Prevention of HIV infection
Duration	4 years	4 years	4 years	7 years
Efficacy	Failed to show efficacy	Failed to show efficacy	25.2% (not statistically significant)	31.2% efficacy (*p* = 0.04)
Immunological Response	Induced immune responses but failed to prevent infection	Early immune responses promising, but no efficacy	Well-tolerated, but no sufficient immune protection	Partial protection through non-neutralizing antibodies and CD4+ T cell responses
Vaccine Regimen	Four doses of Ad26.Mos4.HIV, final two with gp140	Two doses of ALVAC-HIV followed by four concurrent ALVAC-HIV and MF59-adjuvanted bivalent gp120 (subtype C)	Four doses of Ad26.Mos4.HIV, last two with clade C gp140	Four doses of ALVAC-HIV and two doses of AIDSVAX gp120
Geographic Focus	Americas, Europe	South Africa	Sub-Saharan Africa	Thailand
Challenges	Lack of broad, durable immune response in high-risk populations	High HIV incidence overwhelmed vaccine potential	High-risk population and geographic diversity of HIV strains	Specific HIV clades and maintaining durability of protection
Mechanistic Insights	Mosaic immunogens targeting multiple HIV strains but insufficient breadth of immune response	Insufficient antibody and T-cell responses to prevent infection	Further immunological correlates research required	Partial protection linked to non-neutralizing antibodies and T-cell responses
Lessons Learned	Difficulty achieving sterilizing immunity in diverse populations	Need for broader neutralizing antibody responses	Insights for future vaccine development efforts	Combination of humoral and cellular responses may be important for protection
Innovations in Trial	Use of mosaic immunogens targeting global HIV strains	First trial with ALVAC-HIV and gp120 in high-risk settings	Use of Ad26 vector with gp140 protein in Sub-Saharan Africa. Recruited participants from under-represented high-risk groups and engaged closely with local communities.	First trial to show modest efficacy in preventing HIV
Post-Trial Insights	Broad immune responses needed for future vaccine strategies	Viral pressure overwhelmed vaccine’s potential efficacy	Further analysis of immune correlates for protection	Confirmed non-neutralizing antibodies played a key role in modest efficacy
